# Supplementary material for: Are carbon emissions trading and green financial instruments synergistic? -Comprehensive quantitative research based on content analysis
Source: PLoS One. 2024 Mar 7;19(3):e0298601. doi: 10.1371/journal.pone.0298601 (PMC10919601; doi:10.1371/journal.pone.0298601)
Supplement: S1 Data — (ZIP) [file pone.0298601.s001.zip › Data/地级市Z_F工作报告.zip!/╕ú╜¿/─╧╞╜╩╨/└┤╫╘╣1⁄2╓┌║┼í╛┬φ┐╦╩2╛▌═°í┐_2012.docx]

各位代表：

现在，我代表南平市人民政府向大会报告政府工作，请予审议，并请市政协各位委员和其他列席人员提出意见。

一、2011年和本届政府工作回顾

2011年，在省委、省政府和市委的正确领导下，我市各级政府 深入贯彻落实科学发展观，主动融入海西发展大局，全力打好“五大战役”，突出抓好五项重点工作，经济社会发展持续向好，基本完成了市三届人大七次会议确定的年度目标任务，实现了“十二五”良好开局。预计2011年全市生产总值突破800亿元，增长13%（按原口径）；财政总收入77.39亿元，增长25.33%，其中地方级财政收入48.68亿元，增长26.15%；规模以上工业总产值突破1000亿元，增长25%（按原口径）；农林牧渔业总产值320亿元，增长6%；全社会固定资产投资700亿元，增长18%；验资口径实际利用外资7793万美元，增长10%；实际利用内资80亿元，增长29%；外贸出口11.5亿美元，增长26%；旅游接待总人数1519万人次，旅游总收入181.6亿元，分别增长15.2%、16.3%；社会消费品零售总额303.9亿元，增长16%；居民消费价格总水平上涨5.1%；城镇登记失业率3.45%；城镇居民人均可支配收入19585元，增长13%；农民人均纯收入7840元，增长16%；人口自然增长率控制在7‰以内；年度节能减排指标可以完成。一年来主要工作和成效是：

——“五大战役”取得成效。着力突破制约经济发展的瓶颈，多方拓展融资渠道，成立南平农村商业银行，与国家开发银行、农业发展银行签订战略合作协议，争取地方政府债券8亿元，发行企业债券10亿元，通过BT、BOT方式融资24.5亿元；改进征地拆迁工作方法，把维护群众合法利益放在首位，做到公开、公平、公正，依法依规推进和谐征迁，确保“五大战役”顺利开展。重点项目建设有效推进，实施项目230项，完成投资217.9亿元，占年计划103.9%，一批重大基础设施、重点产业和社会事业项目扎实推进，竣工投产和开工项目分别达86项、36项，其中宁武高速公路机场快速通道段竣工通车，龙浦高速公路基本建成，京福高速铁路南平段加快建设。新增长区域建设有序展开，武夷新区完成投资198亿元，占年计划113%；城市总体规划上报省政府，城市规划展示馆、青少年活动中心竣工，新区产业平台、将口交通枢纽、城市快速通道、园区交通路网等配套基础设施加快建设，新区供电区划转调整顺利完成。城市建设亮点纷呈，完成投资86.35亿元，占年计划122.5%；南平中心城市剑州大桥和建溪大桥、常坑大桥、狮子山隧道等沿江快速通道一期工程建成通车；各县（市、区）城市立面改造、景观整治、道路“白改黑”、缆线下地、广告清理、公园建设、公厕改造、违章建筑拆除、交通秩序整治等方面工作取得实效，城市形象进一步提升。小城镇改革发展步伐加快，4个省级试点小城镇完成投资31.22亿元，占年计划108%；小城镇总体规划编制全面完成，各专项规划加快编制；城乡建设用地增减挂钩等试点政策得到落实，农村土地整治和“三旧”改造加快推进；10个市级小城镇改革试点工作顺利实施。民生工程成效明显，实施40个为民办实事项目、6个重大社会事业项目和7个其它民生工程项目，共完成投资53.25亿元，占年计划105.8%。

——“三维”项目对接有力。完善招商引资考核奖励机制，成立招商服务中心，提升行政服务中心，办事效率不断提高，投资软环境进一步优化。央企、民企、外企对接力度加大，

对接“三维”项目137项，总投资881.4亿元。积极参加“6·18”项目成果交易会、“9·8”国际投洽会等展会，先后赴北京、香港等地推介招商，成功举办第十四届海峡西岸武夷国际投洽会暨武夷山国际旅游节、第五届海峡两岸茶博会暨武夷山茶节等重大经贸活动，招商引资形成热潮。台湾统一集团等一批大型知名企业与我市深入洽谈合作，国源纺纱及高档面料生产线等一批投资10亿元以上的项目成功落地。

——产业支撑作用增强。机械（装备）制造、冶金建材、林产加工、食品加工、纺织服装等五个传统产业进一步整合提升，产值占规模以上工业总产值80%以上，生物医药、光电等新兴产业加快培育。实施重点产业项目117项，南铝板带三期等一批项目投产。圣农集团、太阳电缆等一批现有骨干企业实现增资扩产，全市产值超亿元企业198家、净增53家，其中超5亿元企业31家、净增4家。福建元力在创业板上市，建瓯青然食品在天津股权交易所挂牌交易，圣农发展定向增发并发行短期融资券。新增5家省级创新型试点企业，新创中国驰名商标2件、福建省著名商标37件、地理标志证明商标1件。武夷山陆地港正式营运，口岸通关进一步提速。武夷山大红袍山庄、度假区立面改造、邵武云灵山景区等一批旅游项目相继建成，新增3家四星级饭店、2个国家4A级旅游景区。闽北经济开发区、南平工业园区和荣华山产业组团完成投资37亿元，引入企业55家，新增工业产值29亿元。松溪经济开发区升格为省级经济开发区，邵武、建瓯等县（市）工业园区拓展步伐加快。

——“三农”基础持续夯实。落实强农惠农政策，推行政策性农业保险，发放农业补贴3.54亿元。竹木、茶果、畜禽等农业主导产业培育壮大，丹桂、油茶、无患子等特色产业加快发展，规模以上农产品加工企业733家、净增23家。建立农业“五新”示范面积50万亩，培训农村各类实用技术人才3.5万人。除险加固水库50座，新增农村饮水安全人口24.8万人，新建农村户用沼气池8000口。完成土地整理3.45万亩，补充耕地2.5万亩，改造标准农田和池塘1.7万亩。完成造林绿化面积123.2万亩，超额完成全年任务。林权登记发证率86.37%、到户率90.64%。加强农产品质量安全监管，在100家龙头企业建立农产品质量安全可追溯管理体系。“南平机制”持续拓展，推进28个省级“三农”服务中心试点建设，下派第八批科技特派员586名。加快中心村建设，103个市级中心村初步完成验收。抓好农村住房灾后集中重建点的小区管理、景观绿化和农民转岗转产工作，一批集中重建点成为中心村镇建设的样板。

——社会事业加快发展。教科文卫等各项社会事业全面发展，实施中小学校舍安全工程和重建重点项目68项，闽北卫校新校区、南平一中江南校区建设和南平四中校园改造工程基本完成，14所公办幼儿园开工建设；浦城、光泽通过省级“双高普九”验收，建瓯一中、顺昌一中实现创建“省一级达标高中”目标。成功举办第四届科技成果交易会，5个县（市、区）通过全国科技进步考核。南平博物馆开馆并向社会免费开放，新建改造40个乡镇综合文化站，建成311个农家书屋，有线数字电视整体转换工作有序展开。深化医药卫生体制改革，全面实施县级医院、乡镇卫生院改造，医疗卫生服务水平持续提升。新建光泽县社会福利中心、13所农村敬老院、50个社区居家养老服务站、10个社区综合服务中心。低生育水平持续稳定，出生人口素质明显提高，出生缺陷发生率下降到9‰以内。社会福利和慈善事业稳步发展，妇女儿童、老龄和残疾人、民族宗教、外事侨务、气象防震、档案方志、老区库区等工作取得新进展。

——民生水平不断提高。就业和社会保障工作得到加强，新增城镇就业2.3万人，农村劳动力转移就业5.2万人。全民基本医保制度初步建立，新型农村养老保险全面铺开，7个县（市、区）开展城镇居民养老保险试点，企业退休人员基本养老金标准提高，发放社会保障卡215万张。保障性安居工程开工建设20779套、竣工9697套，超额完成全年任务。完成“造福工程”搬迁28518人。落实稳定食品价格措施，市场物价保持平稳。“平安南平”建设持续深化，顺昌、政和平安先行县获得省上命名。道路交通、食品安全等重点专项整治深入开展，安全生产形势总体平稳。落实节能减排责任制，重点流域、重点行业和工业园区污染治理力度加大。国防动员、民兵预备役工作取得新进展，新一轮全国双拥模范城创建通过考评。

——政府自身建设得到加强。自觉接受人大及其常委会的法律监督、工作监督和政协的民主监督，依法执行人大决议，支持政协参政议政，广泛听取民主党派、工商联、人民团体和无党派人士的意见和建议，认真办理人大代表议案和政协委员提案，满意率均为98%。积极推进依法行政，出台重大决策和重大事项听证办法，全面推行政府信息公开，加强电子政务建设，385项审批业务实现网上办理。深入一线协调解决问题，政府服务基层、服务企业、服务群众的能力不断提升。积极推进政府机构改革，市县乡三级政府机构改革基本完成，撤销市本级事业单位33家。推动事业单位人事制度改革和用人方式转变，逐步完善公务员管理制度。严格落实廉洁自律各项规定，廉政建设和反腐败工作得到加强。

各位代表，2011年我市各项工作任务的完成，标志着本届政府实现了市三届人大一次会议确定的主要发展目标。过去五年，在省委、省政府和市委的正确领导下，本届政府坚持以邓小平理论和“三个代表”重要思想为指导，深入贯彻落实科学发展观，自加压力，真抓实干，攻坚克难，负重奋进，实现了全市经济社会又好又快发展。

五年来，我们抢抓机遇融入海西发展大局，武夷新区纳入全省战略。站位海西全局审视闽北的优势、作用和前景，持续提升发展思路，全面展开“一点一线一重心”建设，逐项出台政策，逐项推进落实，海西绿色腹地的地位和作用进一步凸显。着眼于打造闽北发展新增长极，以闽北经济开发区为基础，积极谋划武夷新区建设，发展规划获省政府批准，武夷新区被列为全省十大新增长区域之一，南平科学发展、跨越发展迈入新阶段。加快城镇化进程，南平中心城市和县域城市一批道路桥梁、立面改造、园林景观等项目相继建成，小城镇和中心村镇加快建设，城乡人居环境明显改善，发展空间有效拓展。

五年来，我们坚定不移加快经济发展步伐，综合经济实力明显增强。积极应对国际金融危机，有效克服暴雨洪灾、冰冻灾害等不利影响，保持了经济社会持续较快增长。与2006年相比，2011年全市生产总值、财政总收入实现翻番，规模以上工业总产值、全社会固定资产投资均翻一番半。“突出工业、突破工业”取得实效，4个重点产业产值超百亿元，6家企业产值超20亿元，创建7个省级工业园区。农林牧渔业总产值年均增长5.8%，增幅居全省首位。旅游发展步伐加快，旅游总收入突破180亿元。经济结构进一步优化，三次产业结构调整到21.4∶44.1∶34.5，二产比重提高8.4个百分点。

五年来，我们全力以赴推进基础设施建设，对外战略通道初步构建。累计投入资金305亿元，建成浦南、武邵高速公路和宁武高速公路机场快速通道段，4条高速公路在建，通车里程从2006年的76公里上升到2011年的439公里，居全省前列；3条高（快）速铁路开工建设，全市建成、在建、规划建设的高速公路和铁路均突破1000公里。武夷山新机场前期工作取得新进展。电力网架结构进一步提升，首座500千伏变电站投入运行，电力装机容量新增258万千伏安。

五年来，我们持之以恒保护闽北青山绿水，生态环境质量持续优化。在推动发展的同时，更加重视生态保护。率先在全省开展林木采伐量削减工作，年砍伐量从2006年的244万立方米下降到2011年的180万立方米。实施“四绿”工程，造林绿化228万亩，加强生态公益林管护，森林覆盖率71.14%。深入推进重点流域水环境综合整治，每个县（市、区）都建成并投入使用1座以上污水处理厂，竣工一批城乡垃圾处理项目，水质功能达标率和城市饮用水源达标率均达100%。全面推进生态市建设，南平市国家级生态示范区获环保部命名，省级可持续发展实验区通过验收，生态环境质量保持在全省前列。

五年来，我们千方百计保障民生改善民生，社会和谐程度不断提升。累计完成为民惠民实事120项，城镇居民人均可支配收入、农民人均纯收入年均增长分别为11.6%、12.2%。教育优先发展战略全面实施，文化事业和文化产业加快发展，科技、卫生、体育、广播电视、人口和计划生育等社会事业全面进步，人才工作进一步拓展和加强。革命老区建设取得新进展，5个县（市）被确认为原中央苏区县。加强和创新社会管理，深化社会治安综合治理，“五五”普法顺利完成，10个县（市、区）、95%的乡镇（街道）通过平安建设考评验收，群众对社会治安满意率保持在95%以上。

各位代表，过去五年的成就来之不易。这是省委、省政府和市委正确领导的结果，是人大和政协监督支持的结果，是历届领导班子共同努力的结果，是全市人民辛勤劳动的结果。在此，我谨代表南平市人民政府，向全市人民，向全体人大代表、政协委员，向各民主党派、人民团体、工商联、无党派人士和离退休老同志，向中央、省直驻南平机构和驻军、武警、公安民警，向给予我市灾后恢复重建无私援助的厦门人民，向港澳台同胞、海外侨胞、国际友人，表示衷心的感谢！

在充分肯定成绩的同时，我们也清醒地看到，我市经济社会发展还存在不少问题。主要体现在：经济总量偏小，县域经济总体偏弱；工业化程度不高，主导产业不够突出，产业链不完整，缺少大项目和龙头企业有力支撑；农田水利等基础设施仍然薄弱，抵御自然灾害能力不强；城乡规划、建设和管理水平有待提升，城市辐射带动力不强；社会事业发展和社会管理还存在薄弱环节，安全生产尤其是交通安全仍有不少隐患，生态保护和节能减排的压力增大；一些工作人员思想不够解放，服务理念、工作方式跟不上发展的需要。我们要高度重视这些问题，采取更加有力措施切实加以解决。

二、今后五年主要目标和2012年工作任务

今后五年，是南平科学发展、跨越发展的关键时期，也是提前三年与全省同步建成全面小康社会的决定性时期。我们要积极应对宏观经济形势变化，牢牢把握中央支持海西建设、实施新一轮扶贫开发、统筹区域协调发展和省委、省政府扶持南平发展、武夷新区建设的大好机遇，充分发挥我市生态、空间、资源优势，不断增强要素集聚力、产业承接力、项目吸引力，大力推进新型工业化、城镇化、农业产业化，加快建设充满活力、富裕文明、和谐安康、幸福美好的南平。

根据“十二五”规划和市第四次党代会精神，新一届政府未来五年经济社会发展的主要预期目标是：

——综合实力迈上新台阶。力争实现“一个赶超、两个翻番”，即地区生产总值增幅赶超全省平均水平，2016年全市生产总值突破1600亿元，财政总收入突破150亿元，均比2011年翻一番。

——转型升级取得新成效。实施“三个百亿”工程，培育一批产值超百亿元企业集团、产业集群和工业园区。经济结构进一步调整优化，二产比重突破50%，集约型、创新型、生态型经济架构初步确立。

——基础设施实现新突破。构建大通道，对接港口群，打造大物流，实现县县通高速公路、铁路，基本形成闽浙赣边界重要交通枢纽，电力、燃气等能源保障能力进一步提升。

——城乡建设展现新面貌。围绕山水相融、城景相依的组团式园林城市建设和新农村建设，提升城乡规划水平，完善基础设施和公共服务配套，强化城乡管理，增强集聚功能，城镇化率达到59%。

——生态建设彰显新优势。生态优势充分发挥，经济增长和资源环境承载力更趋协调，节能减排各项指标全面完成，森林覆盖率达到73.54%，生态市基本建成，生态环境质量保持全国全省前列。

——人民生活达到新水平。城乡居民收入大幅增加，社会保障体系不断健全，群众健康水平显著提高，基本公共服务体系覆盖城乡，社会更加和谐稳定，发展成果共建共享，人民群众幸福指数全面提升。

2012年是新一届政府履行职责的开局之年，我们要高举中国特色社会主义伟大旗帜，以邓小平理论和“三个代表”重要思想为指导，深入贯彻落实科学发展观，认真贯彻党的十七大和十七届三中、四中、五中、六中全会及中央经济工作会议和省第九次党代会、省委九届二次全会精神，围绕市第四次党代会确定的“一个赶超、两个翻番、三个提升”目标，继续打好“五大战役”，全力做好稳增长、控物价、调结构、惠民生、抓改革、促和谐各项工作，在更高起点上推进南平科学发展、跨越发展。

今年全市经济社会发展的主要预期目标是：地区生产总值增长12%以上，力争达到950亿元，增长13%；财政总收入增长15%以上，力争达到90亿元、增长16.3%，地方级财政收入增长15%以上；规模以上工业总产值增长20%；农林牧渔业总产值增长6%；全社会固定资产投资增长18%以上；验资口径实际利用外资增长10%，实际利用内资增长20%，外贸出口增长15%；旅游接待人数和收入分别增长12%和15%；社会消费品零售总额增长16%；居民消费价格总水平涨幅控制在4%左右；城镇登记失业率控制在3.8%以内；人口自然增长率控制在7.6‰以内；城镇居民人均可支配收入增长11%以上，农民人均纯收入增长11%以上；完成单位生产总值能耗、化学需氧量、氨氮、二氧化硫、氮氧化物节能减排任务。上述预期目标体现了速度与质量的统一、发展经济与改善民生的统一，体现了“稳中求进”的总基调和在“稳”的基础上更多地着眼于“进”的要求，在实际执行中力求更好更快。

围绕上述目标，2012年要重点做好以下几个方面工作：

（一）致力打好“五大战役”，保持发展强劲势头

拓展领域和内涵。把农林水利、“四绿”工程、城镇美化、“造福工程”、饮水安全、防灾减灾、旅游等领域的项目纳入“五大战役”实施范围，全年安排重点项目245项，完成投资232亿元，竣工投产项目102个、开工建设97个。武夷新区完成投资200亿元，建成投产5000万元以上项目28个、新开工30个。实施城市建设项目255个，完成投资78.3亿元。实施小城镇发展改革试点项目78个，完成投资28.9亿元。办好50件为民惠民实事。

做深做实项目前期。健全项目储备库管理制度，继续深化产业链规划，围绕产业布局和发展方向，精心策划一批带动力强、辐射面广的重大产业项目、基础设施项目和民生工程项目，切实发挥中央、省上扶持南平发展的政策效应。完善重大项目前期工作机制，推动项目前期工作取得实质性进展，形成储备一批、开工一批、投产一批的项目滚动发展格局。

有力保障战役实施。加大资金、资源和要素保障力度，及时协调解决战役实施过程中遇到的困难和问题。继续鼓励通过发行债券、贷款、BT、BOT等方式，扩大融资规模，满足项目建设资金需求。推广和谐征迁工作法，推进“三旧”改造，落实城乡建设用地增减挂钩试点政策，确保重点项目建设用地。推行模块化施工、标准化管理，做到质量、安全、投资、环保和廉政建设同步推进，打造阳光工程、安全工程、优质工程。

（二）致力发展实体经济，加快转变发展方式

推动产业转型升级。突出抓龙头铸链条，深化重点产业链梳理，积极开展产业链招商，引导产业链向高附加值、高技术含量延伸拓展，加快圣农产业化扩建、南纸整体搬迁技改等一批项目建设，推动传统产业新型化。加快培育生物工程、光电、电子信息、文化创意等产业，推进中海油生物燃气、欧浦登触摸屏生产线等一批项目建设，推动新兴产业规模化。围绕构建大武夷旅游经济圈，积极引进培育旅游综合体，整合旅游资源，打造精品线路，培育“旅游套餐”，新增3个国家A级旅游景区，推动旅游产业从观光型向度假型、养生型拓展。扶持发展现代物流业，加快现代物流园区建设，新建8个仓储物流中心、6个大中型批发市场和3个以上大型商业综合体。加快发展总部经济，大力发展文娱、家政、社区服务等家庭服务业，提升信息服务、服务外包等发展水平。

做大做优产业平台。加快平台基础设施建设，进一步完善园区内道路、供水管网、污水处理、电力等配套设施。南平工业园区重点推进江南产业组团一期、陈坑瓦口启动区、大横绿色产业集中区等平台开发，积极引导中心城区企业退城进园，完成基础设施投资2亿元，新增产值12亿元；闽北经济开发区完成基础设施投资21.8亿元，新增产值10亿元；荣华山产业组团重点围绕打造新型轻纺专业城，完成基础设施投资2亿元，新增产值16亿元。大力推进县域工业园区建设，逐步形成一批各具特色的专业园。

促进企业增资扩产。鼓励支持现有企业运用信息化手段、技术革新等方式，提高劳动生产率，增强产品竞争力。引导有优势、有市场、有潜力的企业，通过上市融资、发行债券、引进战略投资者、与大企业合作等方式，实现快速增资扩产。全年新增产值超5亿元以上企业7家。引导企业自主创新，新建2家省级以上企业技术中心，新增5家高新技术企业和创新型企业。积极支持企业创建品牌，鼓励企业参与国际、国家和行业标准制订。落实扶持小微企业发展、促进工业稳定增长的政策措施，积极帮助企业开拓市场、协调用工、提升管理，促进与大企业协作配套、优势互补、融合发展。

加大招商引资力度。突出“三维”项目对接，主动承接发达地区符合环保要求的产业转移，力求在引进大项目、好项目上实现突破。办好海峡西岸武夷国际投洽会和海峡两岸茶博会，积极参加各种展会，提高利用外资水平。充分发挥工商联和商会组织的作用，加强以商招商，做好“回归经济”文章。注重招商实效，围绕项目的功能布局、投资强度、建设进度、优惠政策等要素开展招商洽谈，对重大项目和捆绑项目实行“一企一策”、“一事一议”，强化招商项目的跟踪落实，提高项目签约率、到资率、投产率。坚持把改善投资软环境作为促进招商引资的基础性工作来抓，着力打造统一、公平、透明、可预见的政策环境，廉洁、高效的行政环境，安全、舒适、整洁的生活环境，为招商引资创造良好条件。

（三）致力强农惠农富农，促进农业高效发展

大力发展现代农业。坚持用工业化理念发展农业，用现代科学技术提升农业，用现代经营方式拓展农业，推动传统农业向现代农业转变。稳定发展粮食生产，提高粮食综合生产能力。调整优化种养业布局和品种结构，加快发展设施农业、农产品深加工和流通业，提升农业综合效益。推动农业标准化、规模化、组织化、产业化生产经营，新增规模以上农产品加工企业30家、地理标志证明商标2件。实施土地整理3万亩，补充耕地1.5万亩，建设高标准农田9800亩。建立农业“五新”示范片（场）100个。实施现代种业工程，建设一批良种繁育生产企业和基地。加强闽台农业合作，建设邵武台湾农民创业园、闽台优质水稻合作示范区。加强重大动植物疫病防控和农产品质量安全检测，提高农产品质量。

加强现代水利建设。抓住国家大兴水利建设时机，主动对接全省大水网规划，建立市级水利投融资平台，着力在农田水利、防洪工程、水库除险加固等重点领域取得突破。争取光泽茶富水库列入国家“十二五”水库建设规划，做好政和昌歧洋、建瓯下林、建瓯炮台峡和光泽西口等中型水库前期工作。继续实施建溪、富屯溪防洪工程，争取闽江上游南平段防洪工程捆绑项目立项。开展4条中小河流治理项目建设，完成49座小型病险水库除险加固任务，加快5个县（市、区）山洪灾害防治工程建设，治理水土流失面积21万亩。建成南平市区第二水源工程。推进邵武大乾、光泽肖家坑、武夷山澄浒水库等水源工程建设，解决农村30万人口饮水安全问题。加强小型农田水利工程设施管护，建设一批大中型灌排泵站、灌区等节水性工程。

多渠道增加农民收入。落实强农惠农富农政策，引导更多的信贷资金和社会资金投向“三农”。积极发展政策性、商业性农业保险，进一步引导“农超对接”、“农企对接”，建立完善龙头企业与农民的利益联结机制。大力开展农业生产经营技能培训，培育有文化、懂技术、会经营的新型农民和农村实用人才队伍，鼓励农民优化种养结构，提高经济效益。拓展延伸农业的文化、旅游、休闲功能，大力发展农村服务业。引导外出务工人员返乡创业，鼓励农民就地就近转岗就业。

持续深化农村改革创新。加强农业科技改革与创新，健全完善农业社会化服务体系。建立乡镇土地流转服务中心，引导和规范农村土地承包经营权流转，发展多种形式的适度规模经营。加快林权证登记发放，扩大林权抵押贷款，推进采伐管理制度改革。完善农村金融服务，规范农民专业合作组织经营行为。加大扶贫开发力度，实施新一轮“造福工程”，整村推进帮扶工作，完成农村贫困群众搬迁6390户26757人。深化拓展“南平机制”，充分发挥科技特派员和农村“六大员”作用，加强乡镇“三农”服务中心和村级综合服务场所建设，进一步完善农村“三资”管理，提高农村公共服务和社会管理能力。

（四）致力强化基础设施建设，持续优化发展条件

构建现代立体交通。建成宁武、松建高速公路，加快建设建闽高速公路，推动邵光、延顺、顺邵和南平联络线高速公路BOT招商工作。建成向莆铁路南平连接线，加快建设京福高速铁路南平段，推进南三龙铁路南平段建设，进一步做好浦建龙梅铁路、衢宁铁路南平段前期工作。加快国道、省道改造工程和农村公路网络化建设。开工建设武夷山机场改扩建工程，争取开工南平直升机通航综合服务基地项目，加快推进武夷山新机场前期工作。

加快能源项目建设。新建220千伏变电站2座，新建、扩建110千伏变电站10座。加快新一轮农网升级改造工程。继续推进邵武火电厂扩建、南平核电、海西天然气管网和西气东输管网项目前期工作。

（五）致力建设武夷新区，拓展区域发展空间

坚持规划引领建设。按照“一城六片”功能布局，最大限度发挥武夷山“双世遗”优势，统筹考虑产业发展、城市建设与生态保护。优化城市规划设计，尽量保留原有山体风貌，留足公共空间，做到显山、露水、透绿。进一步深化规划工作，争取《武夷新区城市总体规划》获批实施，抓紧控制性详规、修建性详规及相关专项规划编制，不断完善规划体系。

加快交通路网建设。加快建设高铁武夷山东站站西大桥、站西大道、快速通道等一批重点工程，推动各组团之间紧凑开发、有序推进，实现辐射带动、联动发展。大力实施新岭片区、南林片区、兴田片区、高铁站前广场、崇阳溪沿岸慢行道路、防洪堤及景观绿化带建设，争取开工建设武夷新区二期防洪工程和污水处理厂、自来水厂等配套设施，为各组团建设打好基础。

促进产业加速集聚。用好用足省政府支持武夷新区产业发展的政策措施，继续争取政策支持，落实我市扶持新区总部经济、招商引资、城镇建设和人才引进等政策措施，加快推动产业发展。将口以北（含将口）片区重点发展旅游休闲、创意软件、现代物流等服务产业，童游片区重点发展光电、机械制造、生物医药、食品加工等新型工业。

（六）致力加强城乡建设，推进区域协调发展

增强南平中心城市集聚力。按照现代城市标准，加快大家厂、安济、夏道、湖尾等城市片区和西芹、南山、来舟等卫星镇的规划建设，引导城区企业退城入园，拓展城市规模，拉开城市框架。加快建设沿江快速通道、205和316国道改线、夏道大桥，建成闽江大桥，加强城市桥梁管护和改造工作。发展公共交通，优化公交线路，强化公共停车场等设施配套，改善城市综合交通环境。动工建设2家四星级以上酒店，引进大型城市综合体，做大做强、做精做美南平中心城市。

培育各具特色县域经济体。加快推进县域城市“大城关”建设，继续实施一批基础性、功能性、产业性项目，引导产业和人口向县城集聚，增强县域城市承载能力。按照县域经济差异化发展的要求，发挥各县（市、区）资源禀赋和比较优势，找准定位，明确方向，突出特色，培育引进龙头企业，重点打造一两个有特色的重点产业，加快县域工业化进程，壮大县域经济实力。支持原中央苏区、革命老区、贫困地区发展，扶持少数民族地区、偏远山区、库区发展。

提升城市规划编制和管理水平。以超前的眼光和先进的理念抓好城市规划，严格规划的实施监管，做好城市轮廓线和重点建筑立面设计，合理控制建筑密度和容积率，促进现代元素与传统文化整合，体现城市特色，提升城市品位。继续推进立面改造、景区整治、道路“白改黑”、缆线下地和广告清理等工作，新增绿地面积5400亩，建成25个污水垃圾处理项目，建设13个宜居城市综合体，推进城市净化、绿化、美化、亮化。加强城市社区建设，完善社区服务功能。强化城市综合执法，大力整治违法违章违规建筑，提高城市综合管理水平。

推进小城镇和中心村镇建设。推进4个省级试点小城镇的规划实施，完成10个市级试点小城镇规划编制。以农村灾后集中重建点为样板，推动小城镇建设与中心镇建设、“造福工程”、高山区整村搬迁等相结合，用好土地增减挂钩政策，加快基础设施建设，完善公共服务配套，发展特色产业，建设新型农村社区，形成一批独具风格的组团式、生态型小城镇。

（七）致力深化改革开放，激活发展内生动力

推进重点领域改革。深化投融资体制改革，进一步整合盘活国有存量资产，组建若干个国有集团公司，构建投融资平台。继续推进国有企业改革，妥善处理遗留问题。拓宽民间投资的领域和范围，加快民营经济发展。深化金融改革，鼓励金融机构在南平设立分支机构，扶持设立法人金融机构和产业投资基金，积极发展村镇银行，加大金融服务实体经济的力度。推进财税体制改革，严格控制出国（境）经费、车辆购置及运行费、公务接待费等一般性财政支出，继续做好公务用车专项清理工作。分类推进事业单位改革，推进交警管理体制调整和综合警务改革。创新人才工作机制，深化用人和分配制度改革，营造留住人才、吸引人才的良好氛围。

扩大对外交流合作。加大“引进来”和“走出去”力度，不断增创开放新优势。大力推进山海协作，积极参与海西二十城市协作区、闽浙赣皖九方经济区、闽东北经济协作区，建立更加紧密的区域合作机制。深入推进南台经贸文化交流合作，发挥旅港、旅澳等海外联谊会作用，加强与国外、港澳台地区经济技术合作，不断拓展深化对外开放领域。优化对外贸易结构，增强出口竞争优势。

（八）致力建设生态市，促进可持续发展

加大节能减排力度。大力推进工业、交通、建筑等领域节能工作，加大重点领域、重点行业、重点企业的污染减排力度，提高能源利用水平，确保完成减排任务。注重源头控制，加强投资项目节能评估和审查，落实落后产能退出机制。加快推进重点节能工程和污染减排项目建设，推广节能环保新技术、新装备、新产品，支持有条件的企业加快节能技改。推行清洁生产，积极发展循环经济和绿色低碳产业。

强化环境综合治理。扎实推进闽江流域水环境综合整治，加强规模化畜禽养殖场污染治理，引导畜禽养殖业有序发展。开展工业园区和重点企业污染整治，加快工业园区废水集中处理设施建设，坚决防止环境污染事故发生。加强城市污水管网和配套设施建设，积极推动城市雨污分流。加强饮用水源地保护，依法整治水源保护区污染源，确保饮水安全。全面推进农村环境连片整治示范工程，继续开展农村“家园清洁”行动，不断改善农村环境。

加强生态环境保护。实施生态市建设总体规划纲要，争取更多的生态补偿政策支持，积极创建国家园林城市、生态乡镇、生态村，全面推进生态市建设。大力开展造林绿化和“四绿”工程建设，调整优化树种结构，逐步提高阔叶林比重。严格落实耕地保护和节约用地制度，加大矿产资源开发整合和保护力度，严厉打击非法采矿行为。加强水资源管理，强化河岸生态地保护，加大河道采砂、弃土弃渣、黏土砖生产监管力度，巩固和拓展生态优势。

（九）致力加快文化发展，推进文化强市建设

构建核心价值体系。坚定中国特色社会主义共同理想，大力弘扬民族精神、时代精神和福建精神，学习

宣传詹红荔、朱邦月等道德模范和先进典型，扎实推进群众性精神文明创建活动，提高人民群众的思想道德和科学文化素质。

实施文化惠民工程。完善县市两级图书馆、博物馆、文化馆等公共文化基础设施，并向社会免费开放。加强县级数字影院建设。推进文化信息共享，加快乡镇综合文化站、社区文化中心、农家书屋、公共电子阅览室、公益性上网场所建设。

培育发展文化产业。推进文化改革创新，大力发展文化产品市场，加强文化遗产、民间艺术的保护和开发，培育武夷茶道、朱子文化、大圣文化等文化品牌。引进培养名家大师，推动武夷山创作基地建设，打造一批精品文化力作。借鉴“印象大红袍”运作机制，加强对台、对外文化交流合作，引进培育重点骨干文化企业，推动文化产业发展。

（十）致力创新社会管理，切实保障和改善民生

完善公共服务体系。继续实施中小学校舍安全和扩容改造工程，完成103所义务教育标准化学校建设任务，新建的23所公办幼儿园全部建成并投入使用。争取武夷山通过省级“教育强县”验收，浦城一中、武夷山一中实现“省一级达标高中”创建目标，南平一中江南校区投入使用，南平职业中专新校区一期工程和南平农校职教中心建成，支持南平高校建设。深入实施科技创新工程，打造一批产业技术源头创新基地、高新技术成果转移转化基地和高层次创新创业人才培养基地，强化对园区和企业的人才服务。实施全民健身工程，精心筹办第二届市运会。落实城乡居民基本公共卫生免费服务、城乡困难居民和儿童特殊病种医疗救助，加强县级医院、精神卫生服务机构达标建设，深化基层医疗卫生机构综合改革，逐步推行村卫生所新农合门诊统筹试点和基本药物零差率改革试点，提高人民群众健康水平。加强人口计划生育工作，实施“生育文明·幸福家庭”促进计划，稳定低生育水平，提高出生人口素质。

强化就业和社会保障。全面落实积极的就业政策，做好大中专毕业生就业工作，新增城镇就业2.3万人，转移农村劳动力就业5.2万人。鼓励劳动力自主创业，加强职业教育和技能培训，培养实用型人才。加快推进以公租房为重点的保障性住房建设，新开工建设保障性住房10000套，完善分配、管理、退出办法。完善城镇职工和城乡居民基本养老保险制度，巩固新农合参合率，提高城镇居民基本医疗保险参保率，落实被征地农民社会保障政策。推进事业单位养老保险制度改革，完善城镇职工基本医疗保险市级统筹。继续推进失业、工伤、生育保险制度，扩大覆盖范围。发展社会福利和慈善事业。实施养老服务工程，建设农村敬老院和社区居家养老服务中心。建设妇女儿童活动中心，促进妇女儿童事业发展。

维护社会和谐稳定。加强和创新社会管理，落实社会稳定风险评估机制，完善大调解工作机制，严格按照“路线图”依法处理信访事项，认真接待群众来信来访，及时解决群众的合理诉求，有效预防和化解矛盾纠纷。深化“平安南平”建设，全面实施“六五”普法，完善社会治安防控体系和公共安全体系，严厉打击各种违法犯罪活动。加强对民间融资的监管，有效防范金融风险。推进新一轮“菜篮子”工程建设，做好重要商品产运销衔接，完善市场调控和价格监管，维护市场价格稳定。加强食品药品安全监管，依法打击制售不安全食品药品的行为。落实“预警到乡、预案到村、责任到人”的防灾减灾工作机制。强化安全生产责任，突出抓好交通安全、校车安全、消防安全和生产安全工作，保持安全生产形势稳定。加强国防动员和民兵预备役工作，提高双拥共建水平。

三、着力打造服务型政府

各级政府要始终坚持以人为本、执政为民的理念，勤政廉政，务实落实，着力提升服务企业、服务基层、服务群众的能力，努力建设全心全意为人民服务的政府。

在解放思想中创新有为。把解放思想贯穿于政府工作的始终，思进思新思变，着力改变传统思维定势和运作方式，学习新知识，掌握新技能，进一步开阔眼界、开阔胸襟、开阔思路，不搞短期行为，不做表面文章，始终保持干事创业的精气神。创新工作体制机制，突破重点难点，夯实基层基础，着力破解项目融资、征地拆迁、招商引资难题，推动各项工作取得更大成效。主动对接《海峡西岸经济区发展规划》，围绕闽北产业布局和发展方向，抢抓发展机遇，致力先行先试，勇于创新创造，用好用足用活各项政策，争取更多的项目和资金支持，拓展作为空间。

在落实责任中提高效能。加快政府职能转变，加强机关效能建设，实行一线工作法，及时协调解决发展中的难题。健全评比激励机制，完善量化考核办法，建立目标倒逼工作机制，强化行政问责，加大治庸力度，提高工作效率。强化工作执行力，对看准的事、议定的事，立说立行、一抓到底，务求实效，以实实在在的发展成果论英雄，引导全市各级政府各部门比质量、比速度、比后劲、比作风、比干劲，营造比学赶超、创先争优的良好氛围。

在依法行政中树好形象。自觉接受人大及其常委会的法律监督、工作监督，主动接受政协的民主监督，虚心接受社会舆论和人民群众的监督。落实依法行政实施纲要，加快行政执法平台建设，推进政务公开，规范行政自由裁量权。深化行政审批制度改革，进一步减少和下放审批事项。积极推行电子政务，完善网上审批系统。加强政府廉政建设，强化审计监督和行政监察，加大建设工程项目招投标、经营性土地招拍挂和政府采购等重点领域和关键环节的监督力度，坚决纠正损害群众利益的不正之风，树立为民、务实、清廉的政府形象。

各位代表，我们要紧密团结在以胡锦涛同志为总书记的党中央周围，在省委、省政府和市委的正确领导下，凝聚全市广大干部群众的智慧力量，解放思想，坚定信心，开拓进取，奋力拼搏，扎实推进南平科学发展、跨越发展，为加快建设充满活力、富裕文明、和谐安康、幸福美好的南平而努力奋斗，以优异成绩迎接党的十八大胜利召开！
